# Job Demands and Resources, Mindfulness, and Burnout Among Delivery Drivers in China

**DOI:** 10.3389/fpsyg.2022.792254

**Published:** 2022-03-18

**Authors:** Congcong Zhang, Shannon P. Cheung, Chienchung Huang

**Affiliations:** ^1^Department of Youth Movement History, China Youth University of Political Studies, Beijing, China; ^2^School of Social Work, Rutgers, The State University of New Jersey, New Brunswick, NJ, United States

**Keywords:** job demands, resources, mindfulness, burnout, food delivery, package delivery, China

## Abstract

The food and package delivery workforce in China has grown substantially in the past decade. However, delivery drivers face volatile and stressful work conditions, which can give rise to high turnover and burnout. Past research has indicated that job demands and resources (JD-R) significantly predict burnout. Scholars have also found evidence that mindfulness may be a protective factor against negative outcomes like burnout. Using data collected from 240 food and package delivery drivers in Beijing, China, we examined the effects of JD-R on burnout and whether these relations were moderated by mindfulness. Estimates produced by regression analyses indicated that job demands (JD) have significantly positive effects on burnout (*β* = 0.33), while job resources (JR) have a significant negative effect on burnout (*β* = −0.32). Mindfulness significantly moderated the effects of JD and JR on burnout (*β* = −1.64 and − 1.30, respectively). Results suggest that mindfulness is a protective factor for delivery drivers. Practice and policy implications are discussed.

## Introduction

With its rapidly growing e-economy, China has seen considerable growth in the delivery sector over the past decade (CBNData, 2018; State Post Office, 2020; Chan, 2021; Zhao et al., 2021). Between 2019 and 2020, the online food delivery market alone increased 15%, totaling 665 billion yuan (103 billion USD; China Hotel Association, 2021). In 2019, there were more than 7 million food delivery drivers were employed across the country (Wang, 2021). Similarly, by 2018, China had 3 million individuals employed as package delivery drivers, a 50% increase from 2016 (CBNData, 2018). In 2019, alone, over 60 billion package deliveries made, drawing in a revenue of over 700 billion yuan (108.5 billion USD) and accounting 7.6% of China’s 2019 GDP (State Post Office, 2020). These expanding markets and labor forces come with numerous work demands for delivery drivers (Fang et al., 2017; CBNData, 2018; China Post Express News, 2019; Zheng, 2020; Huang, 2021; Lin and Li, 2021). It comes as no surprise, then, that delivery drivers experience significant burnout (CBNData, 2018; Sun, 2019; Chan, 2021; Wang, 2021). Despite considerable evidence supporting mindfulness’s protective properties against burnout (Hülsheger et al., 2013; Taylor and Millear, 2016; Grover et al., 2017; Guidetti et al., 2019), few studies have incorporated mindfulness into the application of the job demands and resources (JD-R) model to examine burnout in delivery drivers, a burgeoning labor force in China. Thus, this study endeavors to understand how JD-R affect Chinese delivery drivers and whether mindfulness may moderate these relations. Such a study can provide guidance for interventions that might mitigate the occupational stress faced by this rapidly expanding yet vulnerable group of laborers in China.

## Background

It has been well documented that delivery drivers face a significant amount of work-related stress in China. Lin and Li (2021) found that over 95% of food delivery drivers reported experiencing occupational stress, and three-quarters had reported levels of stress that indicated high health risks. Studies have found that the use of algorithmic management tools to monitor driver performance increases the intensity of labor for delivery drivers, and, subsequently, burnout and turnover rates (Sun, 2019; Li and Jiang, 2020; Chan, 2021). For example, Zheng (2020) found that 25% of food delivery drivers had left their position at least once. Work demands not only include long shifts and efficient delivery but also emotional labor, such as the need to engage in emotional regulation and professional behaviors when engaging with customers (Sun, 2019). Yet, for all the work demands involved in this industry, workers’ minimum earnings are not guaranteed (Chan, 2021). Algorithmic management of these platforms allows for flexibility in this line of work, which can seem attractive to some (Zheng, 2020). At the same time, however, when demand for food delivery in a particular location is low, the demand for delivery drivers is similarly low, leading to unpredictable and inconsistent income for the many individuals who rely on this work to support themselves and their families (Chan, 2021). These work conditions have been further exacerbated by the COVID-19 pandemic, which has brought about increasing health risks, job insecurity, and financial instability (Huang, 2021).

Like food delivery drivers, package delivery drivers also experience significant job demands and burnout. Reports indicate that over half of all package delivery drivers work more than 10 h a day, and around one-fifth worked more than 12 h a day (CBNData, 2018; China Post Express News, 2019). As a result, many struggle with work-life balance and have fewer opportunities to engage in rest and leisure (Fang et al., 2017). It follows, then, that studies have found that package delivery drivers experienced low job satisfaction and poor wellbeing, along with high occupational stress, burnout, and turnover (Lin et al., 2018; Xiao, 2019; Zhang, 2019; Li et al., 2020). Zheng (2020) found that 17% of package delivery drivers had left their position at least once. However, many returned to their job due to a lack of work opportunities. This can also be explained by the demographics of delivery drivers, many of whom are migrant workers with low educational attainment (Fang et al., 2017; Zhao and Liu, 2017; Zheng, 2020). The work demands faced by both food and package delivery drivers necessitate the study of potential protective factors that can mitigate their burnout.

The high job stress and negative work outcomes experienced by drivers in China are consistent with findings from various cross-cultural studies (Montoro et al., 2018; Useche et al., 2018; Alonso et al., 2020). For example, Useche et al. (2018) analyzed data from 3,665 Colombian professional drivers and found that about 29% of the experienced high job strain. Likewise, using data from 1,200 Spanish drivers, Alonso et al. (2020) found that about 42% of drivers reported emotional exhaustion at work. In this sample, job stress was positively associated with the number of motor vehicle accidents in the last 3 years. Driving anger appeared to mediate the relations among driving stress, risk predisposition, and traffic sanctions (Montoro et al., 2018).

### The JD-R Model and Burnout

Past literature has applied the JD-R model (Demerouti et al., 2001) to explain how work conditions, classified as either job demands (JD) or job resources (JR), can affect employees’ stress and job burnout (Demerouti et al., 2001; Bakker et al., 2003; Bakker and Demerouti, 2017; Useche et al., 2018; Alonso et al., 2020). JD include physical, social, or organizational job aspects that require sustained physical or mental effort (Demerouti et al., 2001). In the JD-R model, JD are hypothesized to lead to burnout through the “health impairment process” (Bakker and Demerouti, 2017). When an individual must meet the JD of their position or role, they must endure a psychological cost (i.e., exhaustion), which, over time, can deplete their energy and lead to burnout. On the other hand, the job aspects that can support individuals in achieving their work goals, or JR (Demerouti et al., 2001) can reduce burnout through the “motivational process” (Bakker and Demerouti, 2017). In this process, JR reduce the psychological costs associated with JD and reduce the risk of experiencing burnout (Demerouti et al., 2001). Inadequate JR in a work environment can contribute to burnout by leaving individuals without the necessary supports to perform efficiently, which can, in turn, lead to feelings of frustration and withdrawal, as well as disengagement from the job (i.e., burnout).

To date, the JD-R model has been useful for the study of employees’ wellbeing across several lines of work and in many cultural contexts (Bakker et al., 2003; Hakanen et al., 2008; Van der Doef et al., 2012; Kim and Wang, 2018; Van der Heijden et al., 2019; Mäkikangas et al., 2020). Other outcomes of interest include those related to work, such as stress, burnout, work-related health outcomes, and work engagement (Hakanen et al., 2008; Schaufeli et al., 2009; Schaufeli and Taris, 2014; Useche et al., 2018; Alonso et al., 2020). Past findings indicate JD’s major role in developing burnout *via* the health-impairment process, as well as JR’s part in mitigating burnout through the motivation process. In samples using food and package delivery drivers, workload was an especially significant stressor that contributed to burnout (Xiao, 2019; Li et al., 2020). However, at present, the literature on the JD and JR in this particular labor force is limited, as it is only recently that the delivery industry in China has substantially proliferated. Thus, there remains a knowledge gap in the JD, JR, and burnout of delivery drivers and how these relations may differ according to personal characteristics. In this study, we focus on understanding how the relations among JD-R and burnout differ by mindfulness of delivery drivers.

### Mindfulness

The psychological construct of mindfulness and the behavioral health intervention of mindfulness have both been centered in scholarship across a multitude of disciplines (Kabat-Zinn, 2003; Keng et al., 2011; Bartlett et al., 2019; Althammer et al., 2021). The former, as a psychological trait, is defined as a state of conscious awareness that is brought about through purposeful and non-judgmental attention to the present moment (Hanh, 1976; Kabat-Zinn, 1990; Baer et al., 2006). Mindfulness is a multi-dimensional construct, but its two key components can be identified as “mindful attention” and “mindful metacognition.” Mindful attention regulates an individual’s attention by emphasizing an awareness of or alertness to immediate surroundings in the present moment. Meanwhile, mindful metacognition, or “decentering,” can be described as detaching oneself from monitoring reactionary thoughts and feelings. Mindful metacognition is important for individuals to stay non-judgmental of the present moment. At the same time, mindful metacognition does not require that individuals *avoid* having thoughts and feelings in reaction to their immediate surroundings. On the contrary, mindful metacognition encourages that individuals acknowledge their thoughts and feelings as they arise, then “let go” of them (Bishop et al., 2004; Reina and Kudesia, 2020). Mindfulness has trait-like properties in that it varies among individuals and from moment to moment (Bishop et al., 2004; Hülsheger et al., 2013).

Studies on mindfulness continue to proliferate due to mounting evidence of its positive relations with many outcomes, including social and emotional competence (Schonert-Reichl and Lawlor, 2010; Klingbeil et al., 2017) and perceived health and wellbeing (Branstrom et al., 2011; Althammer et al., 2021). Others have suggested that mindfulness can be protective through the regulation of stress reactions in work environments (Roeser et al., 2013; Bartlett et al., 2019; Althammer et al., 2021). In one study, mindfulness had a negative association with emotional exhaustion and a positive association with job satisfaction (Hülsheger et al., 2013). Another study, which collected data from 381 adults with varying levels of educational attainment, found that the different facets of mindfulness could predict certain components of burnout (Taylor and Millear, 2016). “Mindful not reacting” (*p* < 0.05) and “mindful not judging” (*p* < 0.01), for example, were both significantly associated with less emotional exhaustion. These results were expanded upon by Grover et al. (2017), whose analysis of data from a sample of 415 nurses indicated that mindfulness buffered the effects of emotional demands on psychological stress. Together, the literature suggests that mindfulness can be a personal resource that protects against burnout in the JD-R model (Hülsheger et al., 2013; Taylor and Millear, 2016; Grover et al., 2017; Guidetti et al., 2019; Althammer et al., 2021). The ability to pay attention to the present moment in a non-judgmental way can allow individuals to better cope with daily work stressors, reducing their risk of burnout (Bartlett et al., 2019; Smith et al., 2019; Althammer et al., 2021). Thus, mindfulness may have a moderating effect in the relations among JD-R and burnout.

## Objectives and Hypotheses

The objectives of this study are to examine the effects of JD-R on burnout and to investigate the extent of mindfulness mitigate burnout among a sample of Chinese food and package delivery drivers. Based on the JD-R theory and the mindfulness framework (Bakker and Demerouti, 2017; Bartlett et al., 2019; Reina and Kudesia, 2020), we hypothesize the following: (1) JD increase burnout experienced by delivery drivers; (2) JR reduce burnout experienced by delivery drivers; and (3) mindfulness moderates the relations identified in hypotheses (1) and (2). The study’s findings can advance the understanding of how JD-R theory affects delivery drivers and provides evidence of how mindfulness may mitigate burnout among vulnerable delivery drivers in China.

## Materials and Methods

### Data and Sample

The data for the present study came from web-based surveys that were administered to food and package delivery drivers in Beijing, China. Respondents were recruited *via* convenience sampling. The first author engaged with approximately 300 food delivery drivers near large shopping malls with significant concentrations of dining establishments. Recruitment took place between 11 June 2021, and 30 June 2021. A total of 110 food delivery drivers participated in the web-based survey. These drivers were employed by major food delivery platforms in China (e.g., Meituan, Eleme, Dada, and Shansong). After omitting four cases with incomplete answers, the final analytic sample consisted of 106 respondents.

Package delivery drivers were recruited by contacting the main package distribution centers that service Beijing. Fifteen centers, located in the Haidian, Chaoyang, Fengtai and Daxing Districts of Beijing, were selected. These centers housed companies such as S.F. Express, STO Express, YTO Express, ZTO Express, YunDa Express, and Jingdong Logistics. On 6 July 2021, we sent the survey link to package delivery drivers that were employed by the various distribution centers. Approximately 260 drivers received the link. Reminders to participate in the survey were sent to the drivers 7 days and 14 days after the initial invitation. By August 31, 2021, 145 package delivery drivers had completed the survey. After excluding 11 incomplete surveys, the final analytic sample contained 134 package delivery drivers. In total, the final analytic sample contained 240 food and package delivery drivers (106 + 134).

Survey completion time averaged about 12 min. An informed consent process was implemented prior to the survey. Both food and package delivery drivers were informed that the survey was anonymous and that their participation was voluntary. Those who completed the survey received 5 RMB (1 USD). The research protocol was approved by the research review committee at Huamin Research Center in Rutgers University and one of the co-authors’ universities in China.

### Measures

We used the Oldenburg Burnout Inventory (OBI; Demerouti and Bakker, 2008) to measure *burnout*, our main outcome variable. OBI has been verified for psychometric soundness, reliability, and validity in samples that are diverse in occupation, language, and culture (Demerouti et al., 2001, 2003; Halbesleben and Demerouti, 2005). In OBI, burnout is conceptualized as a two-dimensional construct, comprised of exhaustion and disengagement. Exhaustion results from intense and sustained physical, affective, and/or cognitive strain, while disengagement describes the behavior of distancing the oneself from work. Each subscale contains eight items (16 total items). Both subscales contain four positively worded items and four negatively worded items. Response categories ranged from 1 (strongly disagree) to 4 (strongly agree), and responses to positively worded items were reversed-coded so that higher scores would represent greater burnout. Burnout was calculated as the average score of the responses to all 16 items in the instrument. In this study, the Cronbach’s alpha of OBI was 0.79.

We used Lequeurre et al.’s (2013) Questionnaire sur les Ressources et Contraintes Profesionnelles to measure the explanatory variables, *JD* and *JR*. The questionnaire has been used with a sample of Chinese workers and showed high reliability (Cronbach’s alpha above 0.80; Deng et al., 2021). JD were measured by adapting items from the subconstructs of *workload* and *emotional workload*. In QCRP, items measuring *workload* measure the extent to which a respondent perceives that the time needed to meet work responsibilities exceeds the actual amount of time they are given at work. *Emotional workload* describes the effort needed to cope with job-inherent emotions. Job-inherent emotions are emotions that are considered “organizationally desired.” Delivery drivers, for instance, must remain calm under efficiency pressures (e.g., delivering many food orders within a certain time frame) and when faced with challenging customers (e.g., individuals who are angry that their order was incorrect or not delivered on time). Workload and emotional workload were measured by four items each. JR were measured by four items: *relationship with colleagues, relationship with supervisor, support from company*, and *support from customers*. *Relationship with colleagues* and *relationship with supervisor* describe the extent to which an individual perceives that they can receive social support from their co-workers and from their supervisor, respectively. S*upport from company and customers* measures the degree to which respondents feel that they receive support from their company and from customers when they encounter challenges or when their deliveries are late. We selected these specific JD and JR based on a review of the literature and the nature of food and package delivery drivers’ work conditions in China. Response categories followed a 7-point Likert scale, in which 1 represented “never” and 7 represented “always.” Higher scores are interpreted as greater JD or JR. JD and JR scores were calculated by taking the average of all corresponding items’ responses. In this study, the Cronbach’s alpha for JD and JR were 0.84 and 0.70, respectively.

We used Meng et al.’s (2020) Chinese version of the short-form Five Facet Mindfulness Questionnaire to measure *mindfulness*, our moderating variable. This 20-item instrument measures mindfulness as a multi-dimensional construct with five facets: (1) non-reactivity to inner experience; (2) observing; (3) acting with awareness; (4) describing; and (5) non-judging of experience (Baer et al., 2006). *Non-reactivity to inner experiences* is an individual’s ability to stay calm when noticing thoughts and/or feelings that would typically trigger emotional responses. *Observing* is the tendency to notice thoughts and feelings as they arise. *Acting with awareness* is when an individual maintains awareness of the present and simultaneously disattends from potential distractions. *Describing* is the ability to identify thoughts and to label feelings that occur in response to the moment. *Non-judging of experience* is the tendency to consider thoughts and feelings objectively, avoiding value statements or judgments about those thoughts and feelings. In past research, FFMQ has shown high internal consistency and convergent and discriminant relationships with other variables (Baer et al., 2006; De Bruin et al., 2014; Giovannini et al., 2014). More specifically, the short-form version of FFMQ has been found to have high internal consistency and validity (Meng et al., 2020). Responses to items followed a 5-point Likert scale. 1 represented “never,” while 5 represented “always.” We reverse-coded negatively worded items such that responses with higher scores represented greater levels of mindfulness. Mindfulness was calculated by taking the sum of all item responses. Scores could range from 20 to 100. The Cronbach’s alpha for the Chinese version of the short-form FFMQ was 0.90 in this study.

To account for various demographic and socioeconomic characteristics that may affect our variables of interest, we collected data on sex (female = 0, male = 1), age, educational attainment (below high school; high school; and above high school), marital status (0 = married, 1 = never married), and type of delivery (0 = food, 1 = package). Full sample demographics are displayed in Table 1. A majority of the sample (about 80%) was male. The average age of the sample was 35.5. A majority had below high school education (42.9%) or had a high school degree (33.3%). About 42% of sample had never been married. A 55.8% of the sample consisted of package delivery drivers (see Table 1).

**Table 1:** Descriptive statistics of key variables.

**S. No.**		**Mean (SD)**
1.	Burnout [1–4]	2.5 (0.4)
2.	Mindfulness [49–85]	60.5 (6.7)
3.	Jon Demands [1–7]	4.9 (1.3)
4.	Job Resources [1–7]	4.3 (1.4)
5.	Delivery Job Type [%]	
	Food Delivery Driver	44.2
	Package Delivery Driver	55.8
6.	Male [%]	79.6
7.	Age [18–60]	35.5 (10.9)
8.	Education [%]	
	Below High School	42.9
	High School	33.3
	Above High School	23.8
9.	Marital Status [%]	
	Never Married	42.1
	Married	57.9

*N* = 240, Numbers in brackets show ranges of the variable.

### Analytical Approach

Analyses started with descriptive and correlation analyses to examine variable distributions in our sample and bivariate relations among our variables, respectively. Then, we conducted ordinary least squares (OLS) regression analysis to estimate the effects of JD-R on burnout and whether mindfulness moderated these relations, controlling for demographic and socioeconomic characteristics. All analyses were conducted using STATA software 16.0.

## Results

Table 1 presents the descriptive statistics of the variables. On average, the sample reported a burnout score of 2.5 (SD = 0.4) and an average mindfulness score of 60.5 (SD = 6.7). The average JD score was 4.9 (SD = 1.3), and the average JR score was 4.3 (SD = 1.4). In Lequeurre et al.’s (2013) sample of military personnel (*n* = 490), the average JD and JR scores were 3.5 (SD = 1.3) and 5.0 (SD = 1.3), respectively. While these samples are not necessarily comparable due to the different expectations and requirements of delivery drivers and military personnel, the sample averages for our study sample suggest that the delivery drivers had high JD and low JR.

In Table 2, we present the results of Pearson’s correlation analysis. The results were consistent with our first and second hypotheses. JD were positively associated with burnout (*r* = 0.23, *p* < 0.001), while JR and mindfulness were negatively associated with burnout (*r* = −0.26, *p* < 0.001 and *r* = −0.17, *p* < 0.01, respectively). JD-R and mindfulness were highly positively correlated with one another. In further regression analysis, we found that the positive correlation between JD and mindfulness was driven by JR. These results are available upon request.

**Table 2:** Correlation analysis of key variables.

**S. No.**		**1**	**2**	**3**	**4**	**5**	**6**	**7**	**8**
1.	Burnout	–							
2.	Job Demands	0.23^***^	–						
3.	Job Resources	−0.26^***^	0.30^***^	–					
4.	Mindfulness	−0.17^**^	0.15^*^	0.24^***^	–				
5.	Package Delivery Driver	0.12	0.29^***^	0.08	0.06	–			
6.	Male	0.05	0.22^***^	0.16^*^	0.01	0.13^*^	–		
7.	Age	−0.08	−0.33^***^	−0.15^*^	−0.01	−0.36^***^	−0.21^**^	–	
8.	Education – Below High School	−0.02	−0.07	−0.05	−0.05	0.04	0.00	0.07	–
9.	Never Married	0.10	−0.01	0.00	0.09	0.01	0.08	−0.29^***^	−0.16^*^

*N* = 240.

* *p* < 0.05;

** *p* < 0.01;

*** *p* < 0.001.

Table 3 presents the standardized estimates of burnout from OLS regression. JD had a strong and positive association with burnout (*β* = 0.33, *p* < 0.001). On the other hand, JR were negatively associated with burnout (*β* = −0.32, *p* < 0.001). These findings are in line with hypotheses (1) and (2). Mindfulness was also negatively associated with burnout (*β* = −0.16, *p* < 0.05). It was also observed that drivers who had identified that they had never been married experienced greater burnout than those who were married (*β* = 0.15, *p* < 0.05). All other estimates (i.e., delivery type, sex, age, and educational attainment) were not statistically significant. The adjusted R-square of the model was 0.19.

**Table 3:** Regression analysis of burnout.

	*β*	**SE**	***p***
Job Demands	0.33	0.02	^***^
Job Resources	−0.32	0.02	^***^
Mindfulness	−0.16	0.00	^*^
Package Delivery Driver	0.07	0.05	
Male	0.01	0.06	
Age	0.07	0.00	
Education – Below High School	0.03	0.06	
Education – High School	0.07	0.07	
Never Married	0.15	0.05	^*^
			
Adjusted *R*-square	0.19		

*N* = 240.

* *p* < 0.05;

*** *p* < 0.001.

**Figure 1:**
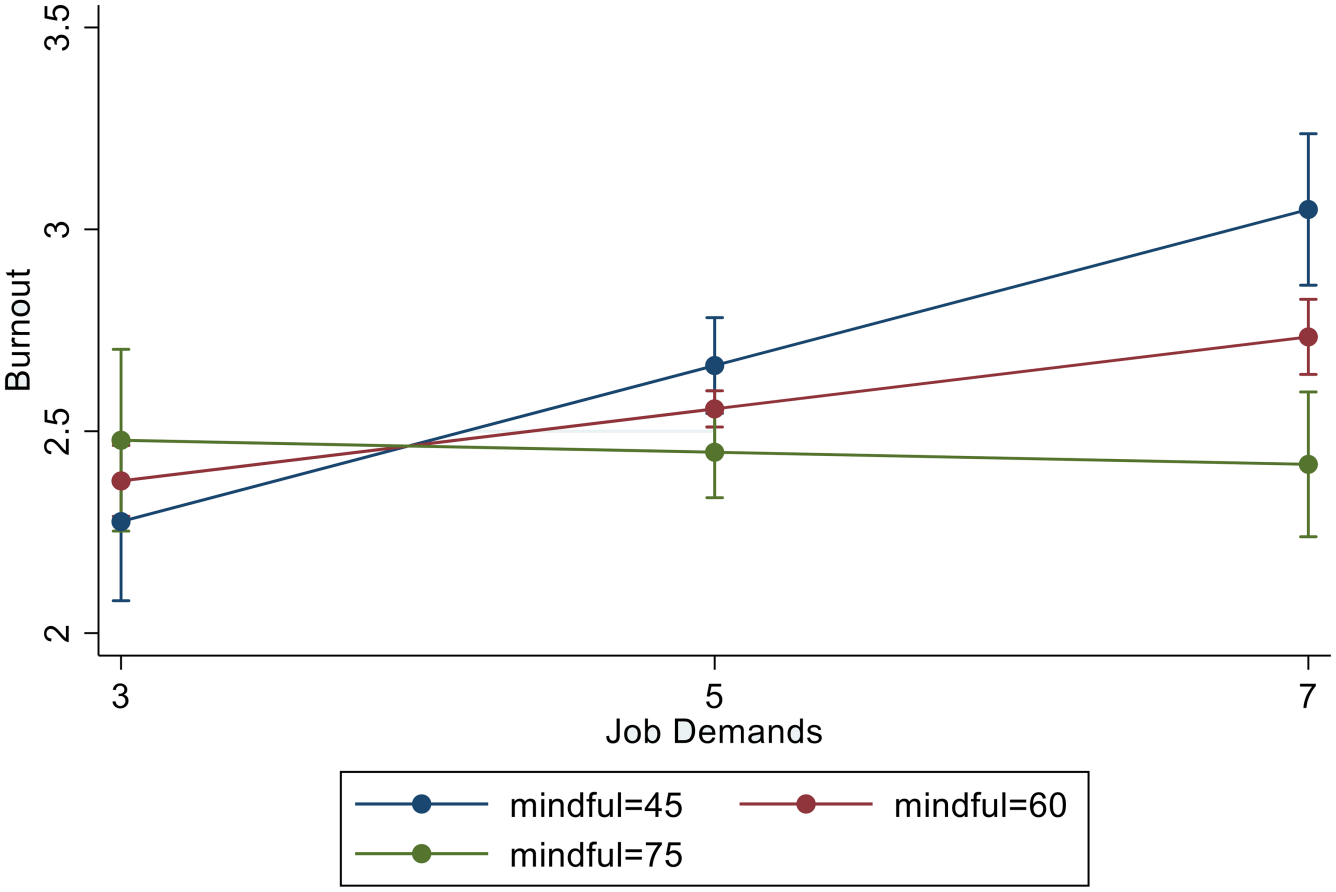
Burnout by job demands and mindfulness.

**Figure 2:**
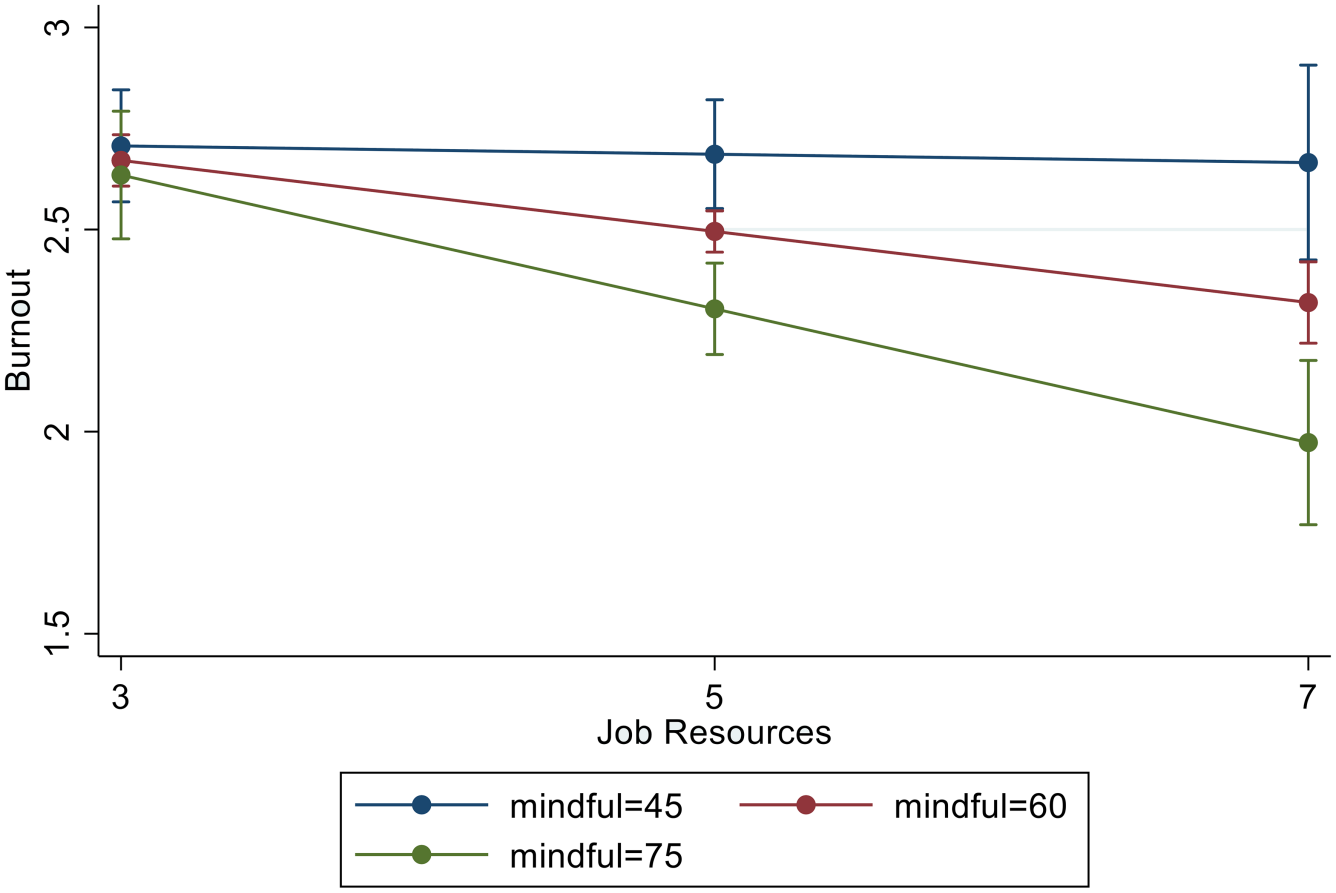
Burnout by job resources and mindfulness.

To test hypothesis (3), which posited that mindfulness would moderate the relations among JD-R and burnout, we added interaction variables—between JD and mindfulness and between JR and mindfulness—to the regression model. To avoid the issue of multilinearity, we ran the regression model twice, each time adding only one interaction variable. The results of these two interaction regressions (i.e., JD^*^mindfulness and JR^*^mindfulness) are presented in Figures 1, 2, respectively. In Figure 1, mindfulness appears to buffer the negative effect of JD on burnout (*β* = −1.64, *p* < 0.001). Likewise, in Figure 2, mindfulness appeared to reduce burnout significantly, specifically when delivery drivers reported high JR (*β* = −1.30, *p* < 0.05).

## Discussion

This study aimed to examine the relations among JD-R and burnout and whether mindfulness moderated these relations in a sample of food and package delivery drivers in China. The results of OLS regression were in line with the hypothesized dual processes of health impairment and motivation by which JD-R affect burnout in our study sample. JD increased burnout *via* the health-impairment process, while JR reduced burnout through the motivation process. The results are in line with those of previous studies conducted in other countries (Bakker and Demerouti, 2017; Useche et al., 2018; Alonso et al., 2020). The magnitude of OLS estimates suggests that both JD and JR have moderate effects on burnout. This raises the need to effectively reduce JD and improve JR among delivery workers, especially given that this labor force tends to experience relatively higher JD and have fewer JR (Useche et al., 2018; Alonso et al., 2020; Li et al., 2020; Wang, 2021). One key facilitator of JD is the use of algorithmic management by food and package delivery platforms, which are designed for profit maximization and efficiency. These management tools fail to consider drivers’ work conditions (Sun, 2019). It is also necessary to increase JR by balancing power among companies, drivers, and customers. As “intermediaries between the company and the public,” delivery drivers’ day-to-day tasks involve high levels of interpersonal contact, which is positively associated with experiencing work aggression (Grandey et al., 2004, p. 399). Further, the common mantra “the customer is always right” is pervasive in service industries and signals the inherent power imbalance between service workers and customers (Hochschild, 1983). Customer aggression can place significant strain on delivery drivers, as they must perform the emotional labor of regulating their emotions and maintaining their composure in the face of a high-stress interaction. Studies have found that, in response to customer aggression and driving stress, workers may adopt a range of coping strategies, including problem solving, escaping or avoiding, driving aggressively, and support-seeking (Yagil, 2008; Montoro et al., 2018; Fombelle et al., 2020). Negative strategies, such as aggressive driving, associated with driving anger, are more likely to lead to negative outcomes, including poor health and work safety (Montoro et al., 2018; Useche et al., 2018), while prevention strategies increased likelihood of positive outcomes (Fombelle et al., 2020). Thus, it is imperative that companies provide support to their drivers, setting realistic expectations for drivers and customers alike. Often, delivery drivers are indiscriminately forced to bear the burden of responsibility in disputes (Sun, 2019; Chan, 2021).

Our findings show that mindfulness can be an effective protective factor against burnout in food and package delivery drivers in China. Not only was the main effect of mindfulness significantly and negatively associated with burnout, but it also significan1tly moderated the effects of JD-R on burnout. For drivers with high JD, mindfulness reduced the negative effect of JD on burnout. Further, mindfulness reduced burnout even more when drivers had high JR. These findings support hypothesis (3). Mindfulness may have had this effect because of its ability to facilitate positive emotion regulation, such that in high-stress situations, individuals can detach themselves from and remain neutral about the present moment. A mindful state can, in turn, allow individuals to make informed decisions about how to cope with a given situation. For example, a delivery driver with high mindfulness may be able make the choice to engage in problem solving or to seek support following an experience with customer aggression (Yagil, 2008; Fombelle et al., 2020). By contrast, if an individual becomes too overwhelmed with reactionary thoughts and feelings, they may behave in accordance, which can place them in unsafe situations (e.g., motor vehicle accidents) and/or lead them to receive disciplinary action (e.g., getting fired or receiving traffic sanctions).

While past studies have shown that JD-R are important predictors of burnout (Purvanova and Muros, 2010; Bakker and Demerouti, 2018; Templeton et al., 2019; Hybels et al., 2020) and that mindfulness may be an important moderator in this relation (Hülsheger et al., 2013; Taylor and Millear, 2016; Grover et al., 2017; Bartlett et al., 2019; Guidetti et al., 2019; Althammer et al., 2021), our study extends this literature by using a sample of delivery drivers, who comprise an emerging and fast-growing labor force in China. This is especially significant, considering the dearth of research on this vulnerable population. Thus, this study provides guidance for both future practice and research.

In applied contexts, the findings suggest that food and package delivery companies in China must reduce drivers’ JD and provide more substantial JR to lower the extent of burnout among their workers. This is also supported by past studies that have similarly reported that drivers tend to experience high occupational stress and burnout but fewer job supports (Lin et al., 2018; Useche et al., 2018; Sun, 2019; Zhang, 2019; Alonso et al., 2020). In addition, given that mindfulness showed significant moderation effects between JD-R and burnout, food and package delivery companies may consider the implementation of mindfulness-based interventions (Bartlett et al., 2019; Guidetti et al., 2019; Althammer et al., 2021). Interventions such as mindfulness-based stress reduction, mindfulness-based cognitive therapy, and mindfulness-based interventions have been found to promote mental health and wellbeing while also reducing burnout (Mellor et al., 2016; Lomas et al., 2018; Slutsky et al., 2018; Bartlett et al., 2019; Guidetti et al., 2019; Suleiman-Martos et al., 2020; Althammer et al., 2021). It is important to note, however, that these studies were conducted in Western cultural contexts, and whether they might have similar results in other cultural contexts, such as in China, is unknown. The results of this study provide support for the utility of future research investigating the extent to which mindfulness-based interventions can reduce burnout in Chinese delivery drivers. So far, a handful of studies (e.g., Hall et al., 2018; Lu et al., 2018; Huang et al., 2019) have found that mindfulness interventions have positive outcomes in Chinese samples, though these studies focused on practice with younger populations. While the mean age of our study sample was 35.5 (range 18–60), these studies focused on children (Lu et al., 2018), adolescents (Huang et al., 2019), and college students (Hall et al., 2018). While it is reasonable to suspect some overlaps in age between our sample and a college student sample, just over three-quarters of the participants (76.2%) in our sample either had high school degree (33.3%) or did not complete high school (42.9%), indicating a significant difference in educational attainment between our sample and those of studies which study college students.

Our study is one of few to center the work experiences of food and package delivery drivers in China, but these findings must be contextualized within a few study limitations. First, the use of a cross-sectional dataset only allows us to estimate associative relations among our main variables. As a result, causal relations cannot be inferred. The use of longitudinal design may better approximate causal relations. Further, our results may be subject to omitted variable bias, as our model may not have included different variables that could affect JD-R, burnout, and mindfulness. The results may also be subject to social desirability bias—and other reporting errors—due to the reliance on delivery drivers’ self-reports for data collection. Triangulating data from multiple sources in future studies may help address this issue. Lastly, the study sample was relatively small and recruited *via* convenience sampling, which limits the generalizability of our results. A future study may build upon these results by using a larger sample that is recruited *via* random sampling.

## Conclusion

This study analyzed data collected from 240 food and package delivery drivers in Beijing, China, to investigate the associations among JD-R, mindfulness, and burnout. The results were consistent with our hypotheses. JD and JR had moderate associations with burnout in opposite directions. Whereas JD and burnout were positively associated with one another, JR and burnout were negatively associated. Mindfulness was protective against burnout, particularly among those respondents with high JD and high JR. These findings expand upon existing research by focusing on Chinese delivery drivers, a rapidly growing labor force that faces high JD and has few JR. The findings also provide support for future work implementing mindfulness-based programs and future studies that evaluate the effectiveness of such interventions to increase mindfulness and protect against burnout in Chinese delivery drivers.

## Data Availability Statement

The raw data supporting the conclusions of this article will be made available by the authors, without undue reservation.

## Ethics Statement

The studies involving human participants were reviewed and approved by Research Review Committee, Huamin Research Center at Rutgers University and the Department of Youth Work Research, China Youth University of Political Studies. Written informed consent for participation was not required for this study in accordance with the national legislation and the institutional requirements.

## Author Contributions

CZ, SC, and CH: conceptualization, methodology, software, validation, formal analysis, and writing—original draft preparation. CZ and CH: resources, investigation, and data curation. All authors contributed to the article and approved the submitted version.

## Funding

This study was supported by the National Social Science Fund of China (No. 21@ZH025) and by China Youth University of Political Studies (No. 666170306).

## Conflict of Interest

The authors declare that the research was conducted in the absence of any commercial or financial relationships that could be construed as a potential conflict of interest.

## Publisher’s Note

All claims expressed in this article are solely those of the authors and do not necessarily represent those of their affiliated organizations, or those of the publisher, the editors and the reviewers. Any product that may be evaluated in this article, or claim that may be made by its manufacturer, is not guaranteed or endorsed by the publisher.
